# Use of Phenobarbital in Delirium Tremens

**DOI:** 10.1177/2324709617742166

**Published:** 2017-11-13

**Authors:** Jeffrey Fujimoto, Jerry J. Lou, Antonio M. Pessegueiro

**Affiliations:** 1 University of California Los Angeles, CA, USA

**Keywords:** alcohol withdrawal, alcohol abuse, delirium tremens, phenobarbital, barbiturates, benzodiazepines

## Abstract

The standard of care for alcohol withdrawal centers on the use of escalating doses of benzodiazepines until clinical improvement is achieved. However, there is no established standard in the care of patients with severe alcohol withdrawal and delirium tremens that is refractory to benzodiazepine therapy. One potential therapy that is gaining traction is the use of phenobarbital, which may be mechanistically superior to benzodiazepines in treating delirium tremens because of its effects on GABA and N-methyl-D-aspartate receptors. The dosing of phenobarbital and its subsequent taper, however, is still unclear and the side effect profile is not well characterized. In this case report, we present the case of a 37-year-old Hispanic male who presented with alcohol withdrawal and subsequent delirium tremens who was treated with phenobarbital with positive clinical response and minimal side effects.

## Introduction

Alcohol withdrawal and subsequent development of delirium tremens (DT) is a potentially life-threatening condition. Appropriate treatment can save lives and avert potentially serious sequelae. In this case report, we discuss the presentation, evaluation, and management of a patient with benzodiazepine-refractory DT with a special focus on the use of phenobarbital.

## Case Presentation

A 37-year-old Hispanic male with a history of chronic alcohol abuse and previous hospitalization for alcohol withdrawal presented with a 1-day history of coffee-ground emesis and tremors. The patient endorsed a 5-year history of alcohol use, reporting consumption of 2 to 3 beers daily and a recent increase to 1 to 2 liters of vodka daily over the past 3 months because of increased stress at work. The patient reported his last drink was the day prior to admission after which he began developing nausea with 3 to 4 episodes of coffee-ground emesis. The patient also endorsed intermittent episodes of bilateral upper and lower extremity tremors. He denied fevers, chills, headache, chest pain, shortness of breath, abdominal pain, focal neurological deficits, seizures, loss of consciousness, hallucinations, hematochezia, or melena. He had no known history of illicit substance use, psychiatric illness, or significant past medical history, though he did endorse 1 episode of alcohol withdrawal treated with chlordiazepoxide 1 year prior to admission.

On physical examination, the patient was hypertensive to 146/98 mm Hg, but had a normal temperature, heart rate, respiratory rate, and oxygen saturation on room air. His abdomen was tender to palpation in the left and right upper quadrants, but without rebound or guarding. He was awake, alert, and oriented to name only. He had bilateral upper and lower extremity tremors that worsened with movement, but otherwise did not have any focal neurological deficits. He also did not have sequelae of chronic liver disease including jaundice, telangiectasis, ascites, or palmar erythema. The remainder of his physical examination was unremarkable.

Initial evaluation included a complete blood count, complete metabolic panel, coagulation studies, serum lipase, blood ethanol level, and toxicology screen. Laboratory studies were significant for an elevated aspartate transaminase (AST) and alanine transaminase (ALT) in an approximate 2:1 ratio (AST 247 U/L, reference 13-47 U/L; ALT 101 U/L, reference 8-64 U/L), elevated serum lipase (252 U/L units; reference 9-63 U/L), and an elevated blood ethanol level (204 mg/dL; reference <15 mg/dL). A right upper quadrant ultrasound demonstrated a normal pancreas and hepatomegaly with steatosis.

Based on the initial presentation and workup, the patient was diagnosed with acute alcohol withdrawal, upper gastrointestinal bleed, alcoholic hepatitis, and alcohol-related pancreatitis. The patient was subsequently admitted to a monitored medical-surgical floor bed and started on our institution’s Clinical Institute Withdrawal Assessment for Alcohol (CIWA) benzodiazepine protocol, as well as intravenous (IV) fluids, thiamine, folate, multivitamin, esomeprazole, and ondansetron. On hospital day 2, the patient continued to have elevated CIWA scores requiring escalating doses of lorazepam. Adjunctive chlordiazepoxide (a longer acting benzodiazepine) at 25 mg orally 3 times daily was added at this point to help achieve better control over his withdrawal symptoms, and the CIWA protocol with lorazepam was continued. The patient had no further episodes of emesis, had no signs of active bleeding, remained hemodynamically stable, and his hemoglobin was unchanged on serial measurements. His initial presentation of coffee-ground emesis was attributed to a possible Mallory-Weiss tear, and endoscopy was deferred in the setting of active alcohol withdrawal. The patient was maintained on a proton pump inhibitor.

On hospital day 4, approximately 75 hours after his last drink, he developed sinus tachycardia (as high as 139 beats per minute), hypertension (as high as 188/112 mm Hg), mild fever to 38°C, diaphoresis, visual and auditory hallucinations, and severe bilateral upper and lower extremity tremors consistent with DT. His withdrawal symptoms became refractory to escalating doses of IV lorazepam, and he was subsequently transferred to the intensive care unit (ICU) for higher level of care. He remained symptomatic despite a total of 25 mg of chlordiazepoxide and 22 mg of IV lorazepam given over the initial 2 hours in the ICU. A decision was then made to initiate treatment with phenobarbital with a loading dose of 500 mg (8 mg/kg based on ideal body weight [IBW]) IV administered over 30 minutes followed by a subsequent maintenance dose of oral phenobarbital tapered over the ensuing 6 days (Table 1). No additional benzodiazepines were given. The patient responded well to treatment with phenobarbital with significant improvement in his tremors, tachycardia, hypertension, and diaphoresis in the first few hours. There were no signs of respiratory depression, and the patient continued to have oxygen saturations above 98% on 2 L/min supplemental oxygen by nasal cannula. The patient did experience hypotension to 75/63 mm Hg shortly after initiation of phenobarbital, but this resolved with IV normal saline boluses.

**Table 1.** Phenobarbital Dosing for Patient.

Hospital Day 4 (Day of Onset of DT and Transfer to ICU)	Hospital Day 5	Hospital Day 6	Hospital Day 7	Hospital Day 8	Hospital Day 9
Loading dose: 8 mg/kg IBW IV over 30 minutes; followed by maintenance dose: 0.5 mg/kg IBW PO 8 hours later	0.5 mg/kg IBW PO BID	0.25 mg/kg IBW PO BID	0.25 mg/kg IBW PO BID	0.125 mg/kg IBW PO BID	0.125 mg/kg IBW PO BID

Abbreviations: DT, delirium tremens; ICU, intensive care unit; IBW, ideal body weight; IV, intravenous; PO, per os; BID, twice a day.

The patient remained in the ICU for 2 days before returning to a monitored medical-surgical floor bed to complete the 6-day phenobarbital taper. He eventually had full resolution of his withdrawal symptoms, nausea, vomiting, and abdominal tenderness. The patient was discharged home on hospital day 9 and he stated he would make an attempt to abstain from alcohol consumption.

## Discussion

This patient experienced a positive clinical response to phenobarbital after developing benzodiazepine-refractory DT. Currently, the standard of care for alcohol withdrawal centers on benzodiazepine therapy until cessation of alcohol withdrawal symptoms occurs or additional pharmacological therapy is needed.^1,2^ The choice of additional therapy, however, is not standardized nor are there well-established regimens in place.^1^ This is particularly important for high-risk patients such as those who have a personal or family history of alcohol withdrawal or DT.^3^ There is growing evidence to suggest that phenobarbital may be an appropriate and effective therapeutic option for alcohol withdrawal, particularly when symptoms are severe.^4,5^ Several protocols employing various dosing regimens of phenobarbital have been reported in the literature.^1,6-8^ In our case report, we used phenobarbital after failed benzodiazepine therapy using a loading dose of 8 mg/kg IBW (500 mg) IV administered over 30 minutes followed by a 6-day oral taper. This regimen yielded a positive clinical response in a high-risk patient without any associated side effects, particularly respiratory depression.

To understand the potential benefit of phenobarbital in the treatment of alcohol withdrawal, it is worthwhile to briefly discuss the effects of chronic alcohol use on the central nervous system. Chronic alcohol use has an inhibitory effect on the brain primarily via 2 mechanisms: activation of GABA_A_ receptors as well as competitive inhibition of N-methyl-D-aspartate (NMDA) receptors, which is the site of action for glutamate, the major excitatory neurotransmitter.^1^ Over time, the brain sensitizes to repeated alcohol use and downregulates inhibitory GABA_A_ receptors and upregulates excitatory NMDA receptors. The elimination of alcohol after long periods of chronic alcohol use thus leads to an inappropriate excitatory response, which manifests in the clinical symptoms of alcohol withdrawal. Additionally, in patients with a history of alcohol withdrawal, there appears to be increased susceptibility to more severe episodes of withdrawal due to the “kindling” mechanism, in which successive insults to the brain during withdrawal leads to increasingly aberrant electrical brain activity, mediated by aberrant NMDA receptor activity.^9^ Lastly, chronic alcohol use has been shown to up-regulate CYP450 enzymatic action in the liver, which may be important for many CYP450-metabolized drugs including those with sedating or activating properties in the central nervous system.^10^

Phenobarbital’s mechanism of action may be better suited for alcohol withdrawal than benzodiazepines, particularly when symptoms are refractory. Benzodiazepines exert their clinical effect by augmenting the frequency of channel opening at GABA_A_ receptors.^11^ Phenobarbital exerts a similar augmenting effect on GABA_A_ receptors by prolonging the duration of chloride channel opening.^3,5^ However, phenobarbital additionally confers the benefit of inhibiting NMDA receptors, which counteracts the “kindling” mechanism and receptor upregulation during chronic alcohol use. As such, the dual mechanism of phenobarbital may confer greater clinical efficacy in the treatment of alcohol withdrawal, particularly in those who are at higher risk due to prior episodes of withdrawal.

The concern with phenobarbital centers primarily on its narrow therapeutic window for respiratory depression relative to benzodiazepines. In our case report, the 37-year-old patient responded well to phenobarbital without signs of respiratory depression. The patient did show signs of sedation, but responded appropriately to commands and never had oxygen saturations below 98% on 2 L/min supplemental oxygen via nasal cannula. However, it should be noted that the patient did not have notable risk factors for respiratory depression, including advanced age, polypharmacy, or history of pulmonary disease. The main side effect observed with this patient was hypotension, which responded well to IV fluid resuscitation with normal saline.

## Conclusion

Phenobarbital as an alternative treatment to benzodiazepines may have significant promise for future therapy, particularly in those at increased risk for severe alcohol withdrawal. There is no current consensus regimen for phenobarbital initiation and tapering for alcohol withdrawal, but our case report demonstrated an effective loading dose of 8 mg/kg IBW (500 mg) IV phenobarbital followed by a 6-day oral taper, which is a higher dosing regimen than many other published studies. This case illustrates the potential benefits of using phenobarbital in severe alcohol withdrawal and will hopefully help inform future clinical practice.

## References

[1] Schmidt KJ Doshi MR Holzhausen JM Natavio A Cadiz M Winegardner JE Treatment of severe alcohol withdrawal. Ann Pharmacother. 2016;50:389-401. 26861990 10.1177/1060028016629161

[2] Dixit D Endicott J Burry L et al Management of acute alcohol withdrawal syndrome in critically ill patients. Pharmacotherapy. 2016;36:797-822. 27196747 10.1002/phar.1770

[3] Sarff M Gold JA Alcohol withdrawal syndromes in the intensive care unit. Crit Care Med. 2010;38(9 suppl):S494-S501. 20724883 10.1097/CCM.0b013e3181ec5412

[4] Martin K Katz A The role of barbiturates for alcohol withdrawal syndrome. Psychosomatics. 2016;57:341-347. 27207572 10.1016/j.psym.2016.02.011

[5] Mo Y Thomas MC Karras GE Jr. Barbiturates for the treatment of alcohol withdrawal syndrome: a systematic review of clinical trials. J Crit Care. 2016;32:101-107. 26795441 10.1016/j.jcrc.2015.11.022

[6] Rosenson J Clements C Simon B et al Phenobarbital for acute alcohol withdrawal: a prospective randomized double-blind placebo-controlled study. J Emerg Med. 2013;44:592-598.e2. 22999778 10.1016/j.jemermed.2012.07.056

[7] Ives TJ Mooney AJ 3rd Gwyther RE Pharmacokinetic dosing of phenobarbital in the treatment of alcohol withdrawal syndrome. South Med J. 1991;84:18-21. 1986421 10.1097/00007611-199101000-00006

[8] Kristensen CB Rasmussen S Dahl A et al The Withdrawal Syndrome Scale for alcohol and related psychoactive drugs: total scores as guidelines for treatment with phenobarbital. Nord Psykiatr Tidsskr. 1986;40:139-146.

[9] Becker HC Kindling in alcohol withdrawal. Alcohol Health Res World. 1998;22:25-33. 15706729 PMC6761822

[10] Balusikova K Kovar J Alcohol dehydrogenase and cytochrome P450 2E1 can be induced by long-term exposure to ethanol in cultured liver HEP-G2 cells. In Vitro Cell Dev Biol Anim. 2013;49:619-625. 23824954 10.1007/s11626-013-9636-y

[11] Perry EC Inpatient management of acute alcohol withdrawal syndrome. CNS Drugs. 2014;28:401-410. 24781751 10.1007/s40263-014-0163-5

